# Patient and economic benefits of psychological support for noncompliant patients

**DOI:** 10.3389/fpsyg.2022.829880

**Published:** 2022-09-15

**Authors:** Phil Reed, Lisa A. Osborne, C. Mair Whittall, Simon Emery, Roberto Truzoli

**Affiliations:** ^1^Department of Psychology, Swansea University, Swansea, United Kingdom; ^2^School of Psychology and Counselling, The Open University, Milton Keynes, United Kingdom; ^3^Swansea Bay University Health Board, Swansea, United Kingdom; ^4^Department of Biomedical and Clinical Sciences, University of Milan, Milan, Italy

**Keywords:** treatment noncompliance, clinical outcome-effectiveness, pelvic floor dysfunction, depression, anxiety, motivation

## Abstract

The current paper provides an overview of treatment noncompliance at various points in the treatment pathway, especially with respect to treatment for Pelvic-floor Dysfunction (PFD). The effects of noncompliance on healthcare are considered, and examples of supporting patients psychologically to increase compliance are discussed. An outline of a method to identify costs of non-compliance, and where such costs most intensely impact the healthcare system, is provided. It is suggested that psychological support is effective in terms of increased compliance and improved healthcare economics. The model is presented for PFD, but the principles developed can be generalised to many aspects of healthcare.

## Introduction

Working on the assumption that the recommended treatment is appropriate, patient compliance exerts a major influence over outcome effectiveness, with noncompliance having deleterious effects both on patient health, and on the economics of healthcare. There are several types of noncompliance, and each type may have specific effects on resources at different points in the treatment pathway (i.e., invitation, completion, and clinical outcome). The current overview presents a possible approach to determining the impacts of these forms of noncompliance on health outcomes and costs by outlining a method identifying noncompliance costs and where these costs most intensely impact the healthcare system. In doing so, it describes examples of the effects of noncompliance on healthcare, and makes suggestions regarding how supporting patients psychologically increases treatment compliance at various points in the treatment pathway.

Traditionally, health economics is employed to aid decision-making about resource allocation. These economic estimates are usually based on quality-of-life adjusted years. One reason for outlining the novel approach described here, is that several problems arise with the more traditional technique; not only are assumptions about the costs of treatment success and failure sometimes questionable, but they may be over-generalised for use in specific healthcare systems. Understanding costs in specific healthcare systems can be improved by in-depth knowledge of patient noncompliance at each point in the treatment pathway, which can predict where resources are being lost, and suggest how to efficiently tackle those problems.

## Compliance

Compliance with treatment is a construct comprising many aspects adversely affecting a cost-efficient and effective healthcare system, including: ‘nonattendance’, ‘nonadherence’, and ‘nonconcordance’ (Turchin et al., 2007). Each of these aspects impacts compliance and outcomes at each of the key points in a treatment pathway: initial attendance, continuation of treatment, and clinical outcome. Failure at each of these successive pathway treatment points has implications for system costs and patient benefits.

### Types of noncompliance

All forms of noncompliance are problematic for all interventions and conditions, and they occur at all points in treatment pathways. *Nonattendance* involves patients not turning up for appointments, and impacts initial take-up and continuation of treatment. *Nonadherence* involves patients turning up for appointments, but not adhering to suggested or agreed treatment regimens. This form of noncompliance impacts treatment continuance and outcome. *Nonconcordance* means patients do not agree with suggested treatments. Nonconcordance will have profound impacts where patients are actively involved in their treatment work.

### Rates of noncompliance

Nonattendance at appointments is estimated at 20%, but this varies depending on the nature of the appointment and condition (Dantas et al., 2018). Self-referral to primary care has a nonattendance rate of 15% (Parsons et al., 2021). Nonattendance rates at referrals into secondary and tertiary care depends on condition and treatment, and varies from 5 to 60% (Binnie and Boden, 2016; Osborne et al., 2017a; Shannon et al., 2018a; Sheridan et al., 2019; Truzoli et al., 2019). Once an appointment has been kept and treatment suggested, nonadherence and/or nonconcordance rates can be high, irrespective of condition or treatment type, and varies from 30 to 75% (Karasu et al., 2000; Partridge et al., 2002; Hauptman, 2008; Basra et al., 2012; Osborne et al., 2016; Truzoli et al., 2017; Yeowell et al., 2018; Shannon et al., 2018b; Peddie et al., 2021).

The specific effects of nonattendance, nonadherence, and noncompliance are difficult to disentangle, but their joint net result can be significant for patient health and healthcare economics. Unfortunately, impacts of noncompliance at different treatment pathway points is not well understood for many disorders, with the possible exception of Pelvic-floor dysfunction (PFD), which may serve as a model in understanding the interactions between noncompliance and the economics of treatment systems.

### Noncompliance in pelvic-floor dysfunction

PFD includes incontinence, prolapse, and sexual dysfunction (Rogers et al., 2018; Milsom and Gyhagen, 2019), and disrupts quality of life and psychological state (Khan et al., 2013; Vrijens et al., 2017). First-line treatment for PFD is pelvic-floor muscle training (PFMT; Khan et al., 2013; Dumoulin et al., 2018; Carty et al., 2019; NHS, 2019; NICE, 2019; Reed et al., 2021), but outcomes and cost are negatively affected by poor patient compliance (Simpson et al., 2019; Reed et al., 2020b).

A nationwide United Kingdom survey (Reed et al., 2020a) found 70–90% initially attending PFMT, with discrepancies between centres. Initial attendance reduces to 50% when waiting time is 16 weeks, or longer (Osborne et al., 2017a), and it is 50–60% for disadvantaged groups (Shannon et al., 2018a). Of those initially invited and attending, not all (from 20 to 60%) complete PFMT treatment. Outcomes for PFMT are reasonably good. Singh et al. (2016) found 51% of PFD patients initially invited for PFMT were successfully discharged from further care for PFD, and similar figures were reported by Hoffman et al. (2017) and Osborne et al. (2021).

**Figure 1:**
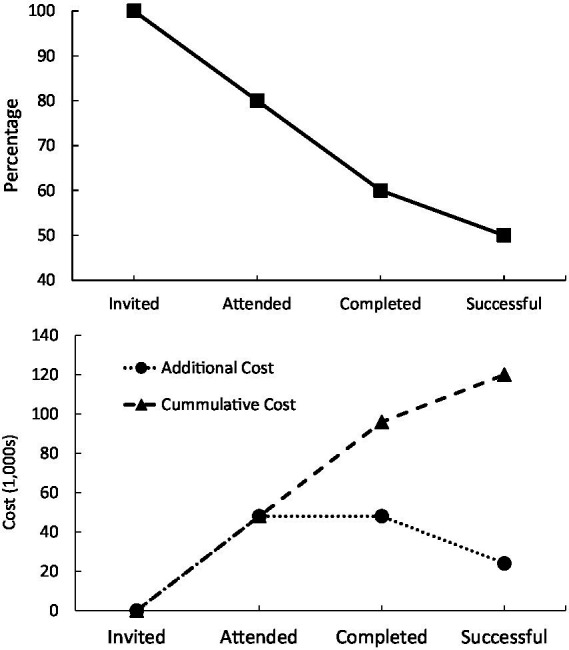
Top panel = example ‘attrition curve’ for pelvic floor dysfunction patients undergoing physiotherapy. Bottom panel = costs of surgical and other procedures per patient in United Kingdom.

The top panel of Figure 1 shows an ‘attrition curve’ for the estimated percentage of patients complying with treatment, at each point in the PFMT pathway, based on the above studies (Singh et al., 2016; Hoffman et al., 2017; Reed et al., 2020a; Osborne et al., 2021). Of 100 patients invited, it is estimated that 80% will attend. Of those initially attending, 70% typically complete PFMT treatment (meaning about 56% of the originally-invited participants complied to this stage). Of this 56%, a sizeable proportion will be discharged from further medical care for this condition, but some will need further treatment. Thus, with 20% who do not initially attend, 24% who initially attend but do not complete, and a smaller proportion (say 10%) who complete but still require treatment, a reasonable figure is that around 45–50% of the initially-invited patients will be discharged from further care for PFD, which is in line with the figures reported by Singh et al. (2016). However, this means that 50–55% could potentially be better supported through their treatment to enhance the outcomes.

One way to examine the economic effects of compliance is to determine the additional healthcare costs that noncompliance incurs to the system. PFMT is relatively cheap, and is estimated to cost: €254 per patient in Holland (Panman et al., 2017), $242–$615 in United states and Canada (Simpson et al., 2019), $124 in Australia (Brennen et al., 2021), and £110 in the United Kingdom (Lamb et al., 2009). However, PFMT is cost-effective only given patient compliance, and noncompliance can necessitate later more expensive surgical treatment. Singh et al. (2016) reported that of the 45% of patients not successful with PFMT in that study, 60% later required surgery. Surgical costs vary depending on what is required, ranging from €25 per min in France to €82 per minute in Sweden, for a 120–240 min operation (Zwolsman et al., 2019). In the United Kingdom costs range between £1,790 for trans-vaginal tape (TVT or mesh) and £4,753 for excision of the uterus (NHS, 2019), and costs in United States range from $3,311 for mid-urethral sling to $5,721 for autologous fascial sling (Wong et al., 2020). Given the variation in surgery costs, and that the cheapest surgical option (TVT) is no longer deemed suitable (Wise, 2018), a median of these surgical alternative costs could (very) conservatively be estimated as £4,000 per patient.

Based on the above estimates of surgical cost, rates of noncompliance as estimated through the attrition curve shown in the top panel of Figure 1 (reflecting 0% attrition at invitation, 20% at initial attendance, 20% at completion, and a further 10% in terms of success), and potential re-referral to surgery (60%), the bottom panel of Figure 1 shows healthcare system costs of additional surgery at each attrition point of the pathway, along with cumulative cost of patients not successful with PFMT. These estimates suggest that, for every 100 patients invited for PFMT, noncompliance and ultimate PFMT failure, results in a £120,000 cost to the system. The largest points of financial loss being initial nonattendance and noncompletion, but this will vary depending on the particular centre.

Such an analysis is an estimate of appropriate additional costs of noncompliance under one set of reasonable assumptions. More importantly, it described a method that could be adapted to individual care systems and treatment domains, adjustable to that particular circumstance, allowing managers to target the point of greatest attrition in their system.

## Treating noncompliance

Many factors are important for understanding noncompliance (Osborne et al., 2017b; Truzoli et al., 2017; Parsons et al., 2021). Several are beyond the control of healthcare system, such as social-economic factors (Shannon et al., 2018a), but some predictors of noncompliance could be targeted with a view to improving compliance and ultimate effectiveness.

Psychological comorbidities are associated with noncompliance and/or poor clinical outcomes for treatment in many medical domains (Cholowski and Cantwell, 2007; Khan et al., 2013; Bennabi et al., 2015; Zanello et al., 2020; Peddie et al., 2021). Supporting some of these patients psychologically, as they undergo medical treatment, may reduce such noncompliance and resource-wastage (Zhang et al., 2007; Truzoli et al., 2017; Osborne et al., 2021). However, for this psychological support to be economically effective, it must improve compliance to the point that the initial medical treatment is working better for higher numbers of patients.

Providing remediation for depression and anxiety is not necessarily straightforward, and these conditions are, themselves, subject to noncompliance and treatment resistance (Gibson et al., 2010; Pigott et al., 2010; Bennabi et al., 2015). That is, patients who are nonconcordant with medical treatment for a physical condition due to their psychological comorbidities also may not comply with treatment directed at addressing those comorbidities. As a result, pharmacological methods of reducing interfering psychological comorbidities alone are unlikely to be effective for this patient group. However, Truzoli et al. (2017) found that an 8-week adjunct group-based Relaxation Response Skills Training programme reduced depression and anxiety, and improved medication adherence, for patients who had not responded to medications. These results suggest that psychological support to augment medical treatment may be effective, even with this group. In fact, beneficial impacts of psychological support have been found for some conditions (Komesu et al., 2011; Al-Sulaiman et al., 2018; Zanello et al., 2020), including pelvic pain (Carty et al., 2019). While these data are encouraging, evidence is sparse regarding how and where such support impacts compliance (and, hence, added healthcare costs) at each point on the treatment path.

### Psychological support for PFMT

With noncompliance estimated between 30 and 60% for PFMT (Singh et al., 2016; Osborne et al., 2017a; Shannon et al., 2018a; Reed et al., 2020a), and high numbers of PFD patients having psychological issues, such as anxiety and depression that are associated with noncompliance and poorer clinical outcomes (Khan et al., 2013; Vrijens et al., 2017; Reed et al., 2021), supporting these issues may improve PFMT outcomes and cost-effectiveness. In fact, targeting the psychological issues may be critical, as several studies have noted that physical pelvic symptoms do not predict compliance and outcome for PFMT (Shannon et al., 2018b).

There are many psychological comorbidities presenting in patients referred for medical treatment, and these psychological comorbidities will vary from condition to condition (Persoons et al., 2005). However, fundamental across many physical conditions are anxiety and depression. These can result from the medical condition, from other psychological conditions also present for the patient, or as a result of other life events quite independent of the medical condition. Irrespective of their origin, they will impact many aspects of functioning, such as memory, motivation, and sensitivity to positive outcomes (American Psychiatric Association, 2013). These aspects of patient functioning are associated with noncompliance.

A variety of broad psychological approaches offer benefits to PFD patients (Komesu et al., 2011; Osborne et al., 2016, 2021; Shannon et al., 2018b; Felsted and Supiano, 2019). Such interventions often target relaxation and stress (Shannon et al., 2018b; Felsted and Supiano, 2019; see Osborne and Reed, 2019, for a review), or motivation and health values (Osborne et al., 2021). Such approaches have involved psychoeducation (giving information about the potential impacts of the medical and psychological conditions), mindfulness (developing conscious awareness of the present and disengagement from the past and future), counselling (including person-centred approaches), cognitive behaviour therapy (targeting and restructuring thoughts about particular situations), and relaxation/hypnosis (including progressive muscle relaxation and suggestion techniques).

Within these broad approaches, many specific techniques have been employed to tackle different aspects of the psychological and behavioural comorbidities. For example, coupled with enhancing memory through reminders (Osborne et al., 2017a; Truzoli et al., 2019), improving motivation to engage with treatment is likely to promote attendance. The positive effects of stronger motivation-to-change on treatments are well documented for many conditions (Bean et al., 2018; Ankawi et al., 2019; Johansen et al., 2019; Reed et al., 2020b). These results suggest that improving motivation directly (or indirectly through reducing depression; see Carty et al., 2019) may benefit compliance. Given this, both counselling and motivational interviewing may be helpful in this context (Osborne et al., 2016, 2021; Bean et al., 2018).

Depression reduces the likelihood of outcomes being valued (American Psychiatric Association, 2013), and valuing outcomes is associated with better compliance and clinical improvements in PFMT (Osborne et al., 2017b; Reed et al., 2020c). Valuing some activities and outcomes—notably in family, work, spirituality, and health domains—has been found to predict attendance at PFMT sessions (Osborne et al., 2017b). However, only holding these values because they are personally important is associated with improved clinical benefits from PFMT (Reed et al., 2020c). Reducing depression, perhaps through support and counselling (Shannon et al., 2018b; Carty et al., 2019), and improving the strength of personal health values (Osborne et al., 2017b) through values-based approaches, may both be useful.

**Figure 2:**
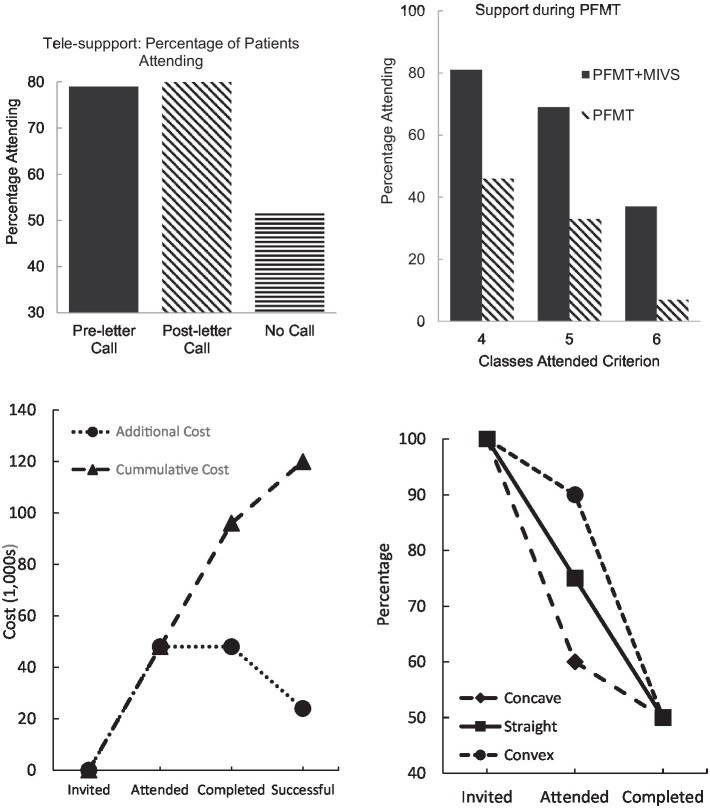
Top left = attendance at initial pelvic-floor muscle training (PFMT) session following brief tele-support (Osborne et al., 2017a). Top right = attendance at PFMT sessions with and without psychological support (Osborne et al., 2016). Bottom left = economic savings from reduced attrition. Bottom right = three type of attrition curve.

A focus on different aspects of the psychological issues may be helpful in reducing noncompliance at specific pathway points (Osborne et al., 2016, 2017a, 2021; Shannon et al., 2018b; Carty et al., 2019; Felsted and Supiano, 2019). One intervention was developed for telephone delivery, and was targeted motivation of patients on a PFMT waiting list (Osborne et al., 2017a; Truzoli et al., 2019). As depression and anxiety reduce behavioural activity and impair memory, a brief support call involving both a reminder of the appointment, and an explanation of the treatment and its possible benefits, was delivered. Osborne et al. (2017a) reported an RCT demonstrating attendance at the initial PFMT sessions improved from 50 to 80% by such a telephone call, delivered either before or after an invitation letter (top left, Figure 2). The initial attendance for the control group was relatively low in this study, but it is in line with studies including many from disadvantaged groups (Shannon et al., 2018a). Other reports have demonstrated text messages can also help bolster treatment compliance for psychologically related somatic distress (Truzoli et al., 2019).

As noted, psychological support improves PFMT attendance and outcomes for those with comorbid psychological problems (Osborne et al., 2016; Shannon et al., 2018b). The results from one RCT of such support (Osborne et al., 2016) are shown in the top right panel of Figure 2, where improvements of 50%were noted. Patients provided with psychological support (MIVS) demonstrated greater improvements in subjective symptoms, quality of life, and anxiety (Osborne et al., 2021; see also Carty et al., 2019; Felsted and Supiano, 2019).

### Improving health economics through adjunct treatment

The above discussed data suggest that supporting patients with comorbid psychological issues can produce up to 60% improved compliance at both initial appointment and during the PFMT programme. Such support also improves clinical outcomes by a similar degree. This allows estimation of cost-savings of such interventions with respect to additional surgery. Noncompliance results in around 50% of noncompliant patients returning for surgery (Singh et al., 2016), at an average cost of £4,000 per patient (see above). If adjunct psychological support reduces noncompliance by 60%, and improves outcomes by a similar amount, then savings on additional surgery can be calculated. These savings are displayed in the bottom left panel of Figure 2 for each treatment point, and cumulatively. Overall, a saving of £60,000 per 100 patients referred can be achieved, based on these estimated figures. These estimates are an example of how such a model can be adapted to individual circumstances.

A saving of £60,000 per 100 patients has been estimated based on data obtained from studies in some centres. This would need to be calculated for each centre. As the number of patients with PFD referred to specialist units usually well exceeds 100 per year (Reed et al., 2020a), then savings will be multiplied by these numbers. These savings should more than cover additional costs of psychological treatment, especially as this support can be provided as part of a group-based PFMT session (Zhang et al., 2007; Osborne et al., 2016, 2021; Truzoli et al., 2017; Shannon et al., 2018b), or can even be delivered virtually by avatar (Andrade et al., 2015).

The data presented in the bottom left of Figure 2 are derived from what could be called a ‘straight’ attrition curve, with equal amounts of noncompliance at each point in the treatment path (in this case 20% at each point). However, other patterns of attrition can also be found, which are shown in the bottom right panel of Figure 2. If most patients drop out of treatment while on the waiting list, then the attrition curve will be ‘concave’. This would suggest that the most cost-effective method of enhancing compliance would be to develop a waiting list support programme. In contrast, if most patients drop out during the treatment, then a ‘concave’ attrition curve will be observed. If this is the case, then attention is needed at providing support during the treatment. If the attrition curve is straight, then both supports may be needed.

## Conclusion

This overview of the effects that noncompliance has on healthcare offered suggestions regarding how psychological support may increase compliance. Moreover, it presented a method to identify the costs of noncompliance, and where those costs most intensely impact particular systems. Where the support is best targeted along the pathway may differ from condition-to-condition, and from healthcare centre to healthcare centre. However, this method of examining the impacts on health economics offers an easy metric to allow such targeting to be individualised, in a manner that more traditional health economics does not.

It is unclear which psychological domains would be most effective to support to improve compliance in different conditions. For example, Truzoli et al. (2017) targeted relaxation and mindfulness for treatment resistant depression and anxiety; and relaxation and hypnotherapy also have been effective with overactive bladder (OAB) patients (Komesu et al., 2011; Osborne and Reed, 2019). In contrast, for PFD, vascular problems, and weight loss, motivation and values have been targeted to promote patient compliance with treatment (Bean et al., 2018; Osborne et al., 2021). This would need to be examined on a condition-by-condition basis, and a mixture of increase relaxation, improved self-efficacy and motivation, and greater focus on valued goals, are common ingredients in any support programme. What is clear is that noncompliance can be addressed psychologically, and that such support will improve the effectiveness and efficiency of healthcare systems.

## Author contributions

PR, LO, CW, SE, and RT: concept and design. LO, CW, and SE: drafting of the manuscript. PR: statistical analysis. PR, LO, CW, and SE: critical revision of the manuscript. RT: supervision. All authors contributed to the article and approved the submitted version.

## Conflict of interest

The authors declare that the research was conducted in the absence of any commercial or financial relationships that could be construed as a potential conflict of interest.

## Publisher’s note

All claims expressed in this article are solely those of the authors and do not necessarily represent those of their affiliated organizations, or those of the publisher, the editors and the reviewers. Any product that may be evaluated in this article, or claim that may be made by its manufacturer, is not guaranteed or endorsed by the publisher.
